# Aeromonas hydrophila Community-Acquired Bacterial Pneumonia With Septic Shock in a Chronic Lymphocytic Leukemia Patient Due to Absolute Neutropenia and Lymphopenia

**DOI:** 10.7759/cureus.23345

**Published:** 2022-03-20

**Authors:** Sachin M Patil, Eric D Hilker

**Affiliations:** 1 Infectious Disease and Critical Care, University of Missouri Health Care, Columbia, USA; 2 Internal Medicine, University of Missouri School of Medicine, Columbia, USA

**Keywords:** lymphopenia, neutropenia, pneumonia, septic shock, bacteremia, aeromonas

## Abstract

*Aeromonas hydrophila* is a gram-negative (GN) bacillus with an opportunistic potential in immunocompromised patients. They are ubiquitary in fresh and brackish water capable of infecting healthy and immunosuppressed patients. Clinical manifestations vary in healthy hosts compared to immunocompromised patients. Community-acquired bacterial pneumonia (CABP) is an infrequent clinical presentation of *A. hydrophila* infection, even in immunosuppressed patients. It is also an uncommon cause of nosocomial and drowning-related pneumonia. Although a rare cause of CABP, the clinical course is fulminant with higher mortality due to lower clinical suspicion. Here, we present an immunocompromised 63-year-old Caucasian male with chronic lymphocytic leukemia (CLL) presenting with acute *A. hydrophila* CABP with septic shock due to absolute neutropenia and lymphopenia.

## Introduction

*Aeromonas hydrophila* is a gram-negative (GN) bacillus ubiquitous in soil habitats and fresh and brackish water. They are facultatively anaerobic, non-sporulating, carbohydrate fermenters and beta-hemolytic on a blood agar culture [1]. Oxidase presence in *Aeromonas *differentiates them from *Enterobacteriaceae* [2]. The annual incidence of *Aeromonas* infections in the United States of America (USA) is 10.6 per million, whereas wound infections account for 0.7% of these cases, with a greater incidence in the younger population (30-39 years of age) [3,4]. In France, the prevalence of *Aeromonas* infections is 1.62 per million [5]. *Aeromonas* septicemia annual incidence in the USA and England is about 1.5 per million [2]. *Aeromonas* is responsible for 2% of the causes of traveler's diarrhea [6]. The risk factors observed with *Aeromonas* infections in patients are age > 65 years, immunosuppression, cirrhosis, and malignancy [7-9]. Clinical manifestations in healthy hosts include diarrhea, posttraumatic infections, and drowning-related pneumonia [2]. In immunosuppressed patients, clinical presentations include bacteremia, sepsis, and extraintestinal infections. The gastrointestinal (GI) tract is the frequent site affected, followed by skin and soft tissue infections (SSTI) [2]. Pulmonary disease is infrequent (<1%) even in immunosuppressed patients, resulting in pneumonia, empyema, and lung abscesses [2,3]. Here, we report a 63-year-old Caucasian male manifestation of acute *A. hydrophila* community-acquired bacterial pneumonia (CABP) with septic shock due to absolute neutropenia and lymphopenia.

## Case presentation

A 63-year-old male with a medical history significant for chronic lymphocytic leukemia (CLL) diagnosed in 2007 on venetoclax, erythema nodosum (EN) on hydroxychloroquine and prednisone, and herpes zoster came to the emergency room with lightheadedness, generalized weakness, and malaise for two days. He denied productive cough, nausea, fever, chills, dyspnea, vomiting, or diarrhea. Over the prior two months, he stopped venetoclax due to recurrent EN exacerbations as it was not well controlled by colchicine, and he was transitioned to hydroxychloroquine and prednisone for the last three weeks. He also received three tapering prednisone courses for EN over the last two months. Clinical examination revealed hypotension of 60/40 mmHg with a temperature of 36.8°Celsius (C), tachycardia of 101 beats per minute (bpm), respiratory rate (RR) of 18/minute, and saturation of 94% on room air. Physical examination revealed him to be weak but well oriented with EN lesions on the bilateral arms, legs, back, and abdomen. Significant laboratory work revealed leukocytosis (neutropenia), thrombocytopenia, elevated lactic acid and procalcitonin, and negative urine legionella and streptococcal antigen, respiratory Biofire 2.1 pathogen polymerase chain reaction (PCR) panel, and coronavirus disease 2019 (Table 1).

**Table 1:** Laboratory results during hospitalization. PCR: polymerase chain reaction, CLL: chronic lymphocytic leukemia, Ig: immunoglobulin, MRSA: methicillin-resistant *Staphylococcus aureus*, HIV: human immunodeficiency virus

Parameters	Values
White blood cell count (3,500–10,500/mL)	29,200/mL (1% neutrophils, 98% lymphocytes) (day 1)
Absolute neutrophil count (>1,500 cells/mm^3^)	292.2 cells/mm^3^ (day 1)
Absolute neutrophil count (>1,500 cells/mm^3^)	2,564 cells/mm^3^ (day 3)
Platelets (150,000–450,000/mL)	109,000/mL (day 1)
Creatinine (0.70–1.20 mg/dL)	1.58 mg/dL (day 1)
Troponin T Gen 5 (≤22 ng/L)	18 ng/L
NT-pro brain natriuretic peptide (0–125 pg/mL)	1,744 pg/mL
Fibrinogen (169–444 mg/dL)	309 mg/dL
Lactic acid (0.5–2.2 mmol/L)	4.8 mmol/L improved to 1.5 mmol/L in the medical intensive care unit (day 1)
Procalcitonin (0.0–0.05 ng/mL)	43.5 ng/mL
Respiratory Biofire 2.1 pathogen PCR panel	Negative (including severe acute respiratory syndrome coronavirus 2, *Bordetella*, *Chlamydia*, and *Mycoplasma*)
Urine analysis	Negative for urinary tract infection
Urine Legionella and Streptococcal antigen	Negative
Peripheral smear	CLL with neutropenia, thrombocytopenia, and macrocytic anemia along with smudge cells and rare and large lymphocytes with prominent nucleoli seen
Tick panel	Negative for ehrlichiosis and anaplasmosis (day 2)
Rocky Mountain spotted fever antibody levels	Indicative of prior infection (IgM < 1:64 and IgG = 1:64) (day 2)
Quantitative IgG levels (700–1,600 mg/dL)	643 mg/dL (day 2)
MRSA nares	Negative (day 3)
CD8 lymphocyte count (145–898 cells/mm^3^)	74.30 cells/mm^3^ (day 4)
CD3 lymphocyte count (550–2,271 cells/mm^3^)	451 cells/mm^3^ (day 4)
CD4 lymphocyte count (365–1,488 cells/mm3)	284.7 cells/mm^3^ (day 4)
Syphilis Treponemal antibody serology	Negative (day 4)
HIV-1 and HIV-2 serology	Negative (day 7)
Acute viral hepatitis panel	Negative (day 7)

**Figure 1:**
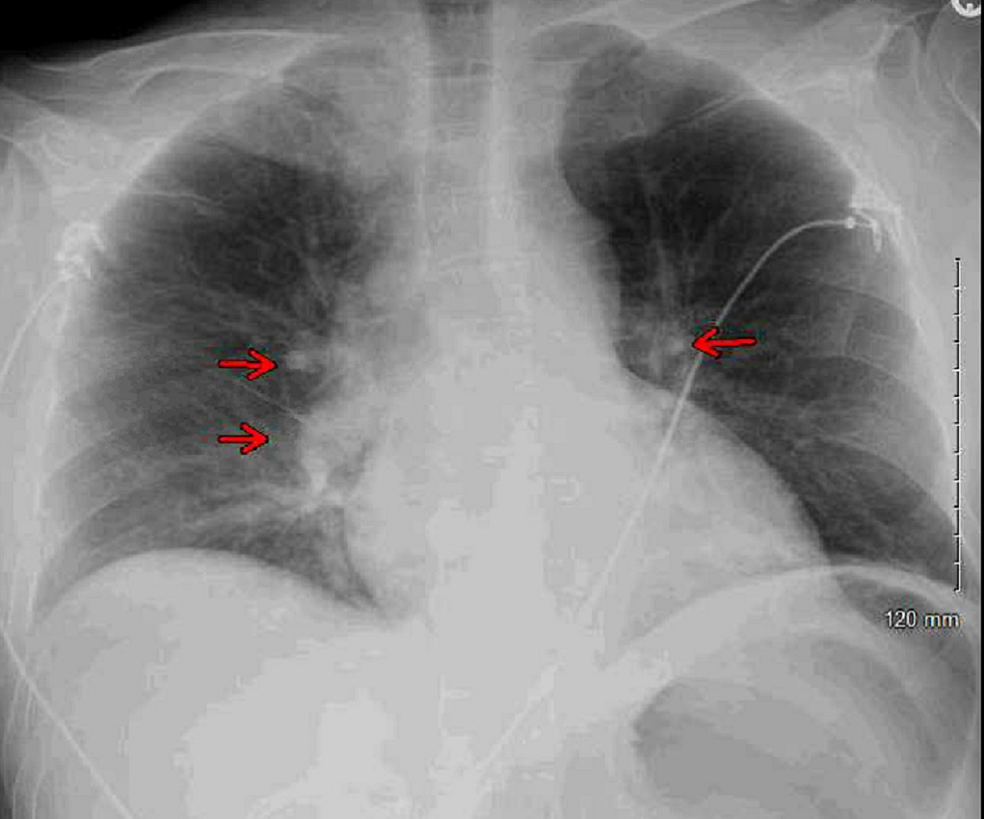
Chest X-ray revealed bilateral perihilar patchy and hazy airspace opacities (red arrows).

Blood cultures and urine cultures were obtained. Chest X-ray revealed bilateral perihilar patchy and hazy airspace opacities (Figure 1).

**Figure 2:**
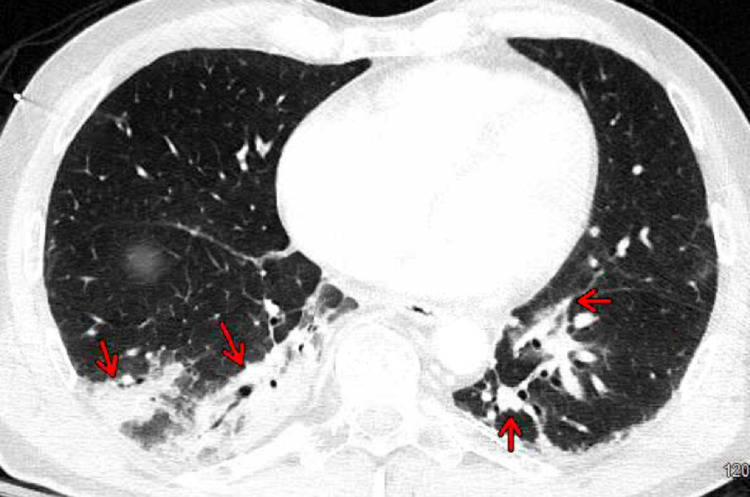
Computed tomography of the chest with contrast axial view revealed bibasilar right greater than left consolidations and ground-glass opacities (red arrows) with subsegmental atelectasis suggestive of pneumonia.

**Figure 3:**
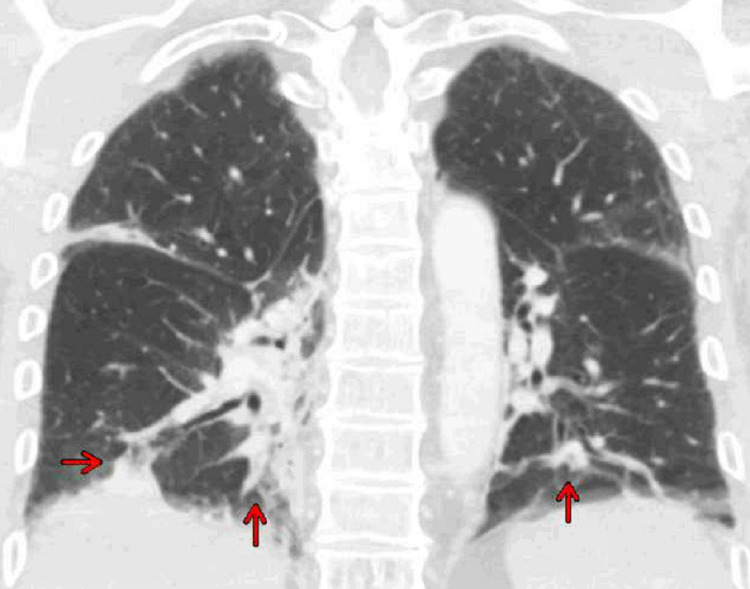
Computed tomography of the chest with contrast coronal view revealed bibasilar right greater than left consolidations and ground-glass opacities (red arrows) with subsegmental atelectasis suggestive of pneumonia.

Computed tomography of the chest, abdomen, and pelvis with contrast revealed bibasilar right greater than left consolidations, ground-glass opacities with subsegmental atelectasis suggestive of pneumonia (Figures 2, 3), and no acute intra-abdominal finding.

As the patient was in septic shock with no productive cough, no sputum cultures or bronchoscopy with bronchioalveolar lavage samples were obtained for culture. Immediately, intravenous (IV) fluids 3 liters (L) normal saline were given, followed by initiation of hydrocortisone (100 mg every eight hours) vasopressors (norepinephrine, vasopressin, and epinephrine), which improved blood pressure to 100/71 mmHg. Venetoclax and prednisone were discontinued. Empirical antibiotics IV vancomycin was administered, and the patient was transferred to the medical intensive care unit (MICU).

In the MICU, clinical examination revealed a well-oriented patient with no new findings, and vital signs revealed a blood pressure of 100/71 mmHg, tachycardia of 108 bpm, tachypnea of 25/minute, temperature of 37.7°C, and saturation of 94% on 4 L nasal cannula. Community-acquired bacterial pneumonia was suspected, and piperacillin-tazobactam was added to vancomycin and doxycycline for suspected tickborne illness. Repeat laboratories revealed improving lactic acid, and vasopressors were continued for target mean arterial pressure ≥ 65. Peripheral smear confirmed CLL with no new findings. On day 2, the blood cultures returned positive for GN rods, and the tick panel returned negative. Blood pressure and creatinine improvement were followed by weaning epinephrine, vasopressin, doxycycline, and oxygen supplementation. Laboratory results revealed hypogammaglobulinemia (Table 1), and repeat blood cultures were negative. He was weaned off norepinephrine on day 3, and the hydrocortisone dose was decreased by half. Laboratory results showed an improved absolute neutrophil count (ANC) to 2,000 cells/mm^3^. Transthoracic echocardiogram revealed a normal study with an ejection fraction of 60%. Admission blood cultures returned positive for *A. hydrophila *complex, and antimicrobials were changed to IV ertapenem. On day 4, oral valacyclovir 1 g thrice a day was initiated for left upper lip cold sore. Clinical flow cytometry was suggestive of lymphopenia (Table 1). Based on *A. hydrophila *sensitivity (Table 2), ertapenem was deescalated to IV ceftriaxone 2 g daily, and he was downgraded to the step-down unit.

**Table 2:** Antimicrobial susceptibility of Aeromonas hydrophila complex. MIC: minimum inhibitory concentration

Antimicrobial	MIC interpretation	MIC dilution
Amikacin	Susceptible	≤2
Cefazolin	Resistant	≥64
Cefepime	Susceptible	≤1
Cefoxitin	Susceptible	≤4
Ceftazidime	Susceptible	≤1
Ciprofloxacin	Susceptible	≤0.25
Gentamicin	Susceptible	≤1
Levofloxacin	Susceptible	≤0.12
Piperacillin/tazobactam	Resistant	≥128
Trimethoprim/sulfa	Susceptible	≤20

Over the next four days, serology for human immunodeficiency virus, acute viral hepatitis, and syphilis was negative, and acid-fast bacilli blood cultures returned negative. The dermatology team recommended a hydrocortisone switch to a gradual taper of prednisone over two weeks for EN. The infectious disease team recommended two weeks of IV ceftriaxone 2 g daily to complete therapy for *A. hydrophila* pneumonia with septic shock. Due to lymphopenia and prednisone use, he was also placed on pneumocystis prophylaxis for one month with atovaquone (allergy to sulfa and quinolones). He completed seven days of oral valacyclovir for the cold sore. He was discharged on day 8 and completed his antimicrobials successfully, followed by a negative blood culture after two weeks of therapy.

## Discussion

*Aeromonas* virulence is due to surface components (pili, S-layer, and flagella), secretion system type three, proteases, and cytotonic and cytotoxic enterotoxins. *Aeromonas hydrophila* is notorious for carrying various toxins simultaneously [2]. Compared to other *Enterobacteriaceae*, *Aeromonas* infections in a mice model revealed a potent higher level of pro-inflammatory cytokines [10]. *Aeromonas* bloodstream infection (BSI) cause often includes three species (*A. hydrophila*, *A. caviae*, and *A. veronii*), of which the *A.* *hydrophila* accounts for most cases [9,11]. *Aeromonas* BSI source is often from the gastrointestinal tract, SSTI, or secondary infections. Of septicemic patients, 80% are seen in immunocompromised middle-aged male patients with a community-acquired infection, often in summer [2]. *Aeromonas* septicemia is often monomicrobial and associated with a higher mortality rate of ≥33% [2]. *Aeromonas*-related healthcare-associated bacteremia is frequent in immunosuppressed patients and has a higher fatality [11]. Factors associated with a worse outcome are confusion, cirrhosis, cancer, septic shock, community-acquired or secondary bacteremia, and multiple sets of positive blood cultures, of which only septic shock and active cancer at presentation are independently associated with death [8,11-13]. They are resistant to penicillin, ampicillin, and cefazolin but are susceptible to third-generation cephalosporins, carbapenems, and aztreonam [2]. Rarely, they are capable of extended-spectrum beta-lactamase and metallo-beta-lactamase synthesis, providing broader beta-lactam resistance [2]. In vitro studies indicate quinolones or cephalosporins (third or fourth generation) as the antimicrobial choice in *Aeromonas* bacteremia [11].

*Aeromonas* pneumonia is the most common respiratory complication and has two distinct clinical presentations [2]. The first is associated with trauma, as observed with near-drowning accidents. *Aeromonas​​​​​​​ hydrophila* is the most common cause of pneumonia in drowning patients and is the most serious of all causes [14]. *Aeromonas* pneumonia is possibly due to the aspiration of a large amount of water containing a larger bacterial inoculum that can quickly lead to hemorrhagic necrotizing pneumonia due to its fulminant nature (<24 hours) in a hypoxic lung [15]. The current understanding of this rapid fulminant nature is limited [16]. The second presentation is observed in patients with no respiratory events in the presence of underlying risk factors. Some suspected causes include aspiration, contaminated water exposure, GI source, or secondary infections. Here, blood cultures are often positive, along with cultures from tracheal aspirate, bronchoalveolar lavage, and pleural effusions. Here, the rapid fulminant clinical course lasts less than ≤48 hours [17]. Information from case reports indicates *Aeromonas* pneumonia-related mortality to be very high at 50% [2]. Factors predicting poor outcomes include a shock, cancer, liver cirrhosis at presentation, intensive care unit admission, and mechanical ventilation. In *Aeromonas* pneumonia, the antimicrobials of choice are third- or fourth-generation cephalosporins or quinolones [7].

Our patient was an avid gardener and possibly inhaled contaminated water droplets into his lungs. Although venetoclax use can result in prolonged neutropenia, lymphopenia, mucositis, nausea, vomiting, abdominal pain, sepsis, and pneumonia, our patient was not taking this medication for the last two months [18]. Another explanation is that immunosuppression promotes transient colonization, and gastrointestinal mucosal injury or translocation results in BSI [2,18]. Absolute neutropenia (ANC = 292.2 cells/mm^3^) at presentation and lymphopenia (CD3 = 451 cells/mm^3^) in our patient predisposed him to infections [19]. The precise duration of long-term venetoclax effects is unknown. Our patient did not present with the classical signs of bacteremia or pneumonia. The clinical presentation of *Aeromonas* bacteremia and pneumonia can be nonspecific, and it can be challenging to pinpoint the organism as the cause. This infection needs to be considered in any patient with fresh or brackish water contact or the presence of ecthyma gangrenosum skin lesions (bullae or petechiae due to bacteremia) [2]. *Aeromonas *in respiratory samples should be assessed by clinical evaluation and not considered contaminants [20]. Due to the higher mortality and rapid clinical severe course, it is imperative to start on broad-spectrum antibiotics as in our case.

## Conclusions

An increasing incidence of opportunistic infections in immunosuppressed patients combined with the nonspecific presentation of *A. hydrophila* pneumonia makes it challenging to determine the etiology. An essential part of increasing suspicion about this pathogen is investigating the patient's clinical and occupational history and recreational activities involving water. Increased awareness of this infection among at-risk patients might help them seek medical attention earlier. A lower threshold of clinical suspicion and early antimicrobial therapeutic intervention is perhaps the best way to reduce the high mortality and morbidity in *Aeromonas* pneumonia.
